# Barriers and Facilitators for Mental Health Service Use Among Racial/Ethnic Minority Adolescents: A Systematic Review of Literature

**DOI:** 10.3389/fpubh.2021.641605

**Published:** 2021-03-08

**Authors:** Wenhua Lu, Abigail Todhunter-Reid, Mary Louise Mitsdarffer, Miguel Muñoz-Laboy, Anderson Sungmin Yoon, Lei Xu

**Affiliations:** ^1^Department of Community Health and Social Medicine, School of Medicine, City University of New York, New York, NY, United States; ^2^American Educational Research Association, Washington, DC, United States; ^3^Department of Childhood Studies, Rutgers University, Camden, NJ, United States; ^4^School of Social Welfare, Stony Brook University, New York, NY, United States; ^5^School of Social Work, Nyack College, New York, NY, United States; ^6^Department of Health Education and Promotion, College of Health and Human Performance, East Carolina University, Greenville, NC, United States

**Keywords:** mental health service use, adolescent/youth, racial/ethnic minorities, facilitators and barriers, systematic review

## Abstract

**Introduction:** Mental disorders represent serious public health concerns in the U.S. Compared with Whites, racial/ethnic minority adolescents are more likely to be affected by mental disorders but less likely to use mental health services. This systematic review aimed to summarize factors related to mental health service use among minority adolescents in the U.S. as identified in previous research.

**Methodology:** Following the PRISMA guideline, we systematically searched seven databases for peer reviewed articles related to barriers and facilitators of mental health service use among racial/ethnic minority adolescents.

**Results:** Thirty-two quantitative studies met our inclusion criteria, among which 12 studies (37.5%) sampled mostly Blacks or African Americans, 6 studies (18.7%) focused primarily on Hispanics or Latin/a/x, including Mexican Americans and Puerto Ricans, and 4 studies (12.5%) were mostly Asian Americans (e.g., Chinese, Vietnamese). Based on the socio-ecological framework, 21 studies (65.6%) identified adolescent-related barriers and facilitators of mental health service use, including biological (e.g., age, gender), clinical (e.g., symptom severity), behavioral (e.g., drug/alcohol use), and psychological characteristics (e.g., internal asset) of minority youth. Ten studies (31.3%) identified parents-related factors that influenced minority adolescent mental health service use, including parental perceptions and beliefs, family and parenting issues, and demographic characteristics. Primary factors at the therapist level included ethnic match between patient and practitioner, relationship with healthcare practitioners, and patient-therapist co-endorsement of etiological beliefs. Fifteen studies (46.9%) identified factors influencing minority adolescent mental health service use at the contextual/structural level, including household income, insurance status, and family structure. Lastly, acculturation and school experiences were major factors at the social/cultural level that influence minority adolescent service use.

**Conclusion:** More empirical studies are needed to understand the mechanism underlying minority adolescents' unmet mental health service needs. Culturally competent interventions are warranted to engage minority adolescents with mental disorders into treatment.

## Introduction

Mental disorders among adolescents are characterized by “serious deviations from expected cognitive, social, and emotional development” and include conditions meeting criteria described in the Diagnostic and Statistical Manual of Mental Disorders (DSM), e.g., depression, anxiety, and developmental delays (1, 2). As the transitional stage of vital neurodevelopment between childhood and adulthood, adolescence poses a vulnerable time for the onset of mental disorders due to various physical, emotional, and social changes (3). Globally, 1 in every 6 adolescents aged 10–19 experience a mental disorder (4); in the U.S., mental disorders are impacting up to 50% of youth in their lifetime (5, 6). Although effective treatments are available to help adolescents manage their mental health disorders, most adolescents with mental disorders do not receive proper treatments (7, 8). Untreated mental health disorders can lead to substantial negative health and social consequences, including academic failures, self-injuries, substance abuse, violence, crime, chronic physical disorders, and suicidal behaviors (9–11).

Compared with Whites, racial/ethnic minority adolescents are at greater risk of mental disorders, but less likely to use mental health services. Based on findings from two recent studies, for example, Black and Hispanic adolescents with depression are much less likely than Whites to receive mental health services in general and across different settings, including specialty mental health and general medical settings (7, 8). Further, among all racial/ethnic groups, Asian Americans are least likely to use medication for depression (7). Even if they do receive treatment, minority adolescents are more likely to terminate treatment prematurely. To make sure minority adolescents receive timely treatment and recover from mental disorders, effective interventions are essential to engage and retain them in treatment.

Identifying barriers and facilitators associated with mental health service use constitutes a fundamental initial step for the development of culturally adequate engagement interventions for minority adolescents and families. Barriers are the reasons or obstacles that prevent individuals from seeking, obtaining, or completing mental health treatment, whereas facilitators are those factors that make the process of seeking, obtaining, or completing mental health treatment easier or more likely (12, 13). Although some literature reviews have summarized barriers and facilitators for mental health service use among children and adolescents (12, 14–16), none of them focused on racial/ethnic minority adolescents in the U.S. Given the unique social and cultural contexts that minority adolescents navigate in the U.S., it is important to understand their experiences when seeking mental health services. This systematic review, therefore, seeks to fill this critical research gap by synthesizing recent findings from quantitative studies on barriers and facilitators for mental health service use among racial/ethnic minority adolescents in the U.S.

## Methods

### Search Strategy

Following the PRISMA guideline (17), we systematically searched the following seven comprehensive databases for peer-reviewed articles related to barriers and facilitators of mental health service utilization among racial/ethnic minority youth: Academic Search Premier, CINAHL, MEDLINE, PsychINFO, ERIC, PAIS, and Social Work Abstract. Different combinations of the following search terms were used: youth, adolescent, child or teen; mental, psychological, psychiatric or emotional, depression, anxiety, ADHD; race, ethnic, minority or immigrant; service, therapy, refer, or help-seeking; factor, barrier, or facilitator. These search terms were selected based on librarian and researcher expertise. For different databases, the search was adapted as appropriate. After the key word searches, duplicated articles between and among the databases were identified and excluded by hand.

### Inclusion and Exclusion Criteria

To be included in the review, studies had to (1) be empirically based, (2) be conducted in the U.S., (3) include adolescents aged 10–19, (4) recruit solely or mostly racial/ethnic minorities, and (5) report factors that influenced mental health service use among minority adolescents. Studies that did not target minority adolescents but conducted sub-group analyses to identify race/ethnicity-specific factors related to service use were also eligible. While adolescents are commonly defined as 10–19-year-olds, definitions in the literature vary (18, 19). Therefore, an inclusive approach was employed in this review whereby studies were included as long as they included adolescents aged 10–19, with no lower age limit and an upper age limit of 24 (e.g., 5–15 years, 22 years and under). To make sure we provide the most current evidence to guide intervention development, we restricted our search to studies published after January 1, 2000. Further, to facilitate data synthesis, only findings from quantitative studies or quantitative results of mixed-method projects were analyzed. For quality control purpose, studies that only reported descriptive statistics (e.g., frequency) were excluded from synthesis. Lastly, to facilitate generalizability, studies were excluded that focused exclusively on homeless adolescents or adolescents in child protective services custody, juvenile detention facilities, or foster care.

Based on DSM-V, mental disorder is defined as “a syndrome characterized by clinically significant disturbance in an individual's cognition, emotion regulation, or behavior that reflects a dysfunction in the psychological, biological, or developmental processes underlying mental functioning” (20). As listed in DSM-V, we examined internalizing problems (e.g., depression, anxiety, bipolar disorders), externalizing behaviors (e.g., ADHD, oppositional defiant disorder, conduct disorders), and substance use disorders (e.g., alcohol abuse/dependence, drug abuse/dependence) in this review. A standard definition of mental health service use does not exist to date. Generally, in studies of mental health service use, researchers focus on how people use the formal system of care, including psychiatrists, psychologists, social workers, in-patient psychiatric units, and out-patient mental health programs (21). For this review, we broadly defined mental health service use as referral to, receipt of, and retention in any type of professional services for mental health problems, including clinic-based specialty or outpatient services, school-based services, and emergency or inpatient services.

Screening of the unique studies was conducted at three stages, i.e., title screening, abstract screening, and full-text screening, by two authors independently. Disagreements were resolved by discussing with the first author until consensus was reached. To ensure the search was exhaustive, we further examined reference lists of the most recently published articles that passed the full-text screening for relevant articles that were missing from the original search. The last search was conducted in August, 2020.

### Data Extraction

Data from the reviewed articles were abstracted based on Garrard's matrix method of literature review in health science (22) and previously validated instruments (23, 24). Information extracted from each study included study characteristics (e.g., study design, study setting/area, sample size, instruments used), participant characteristics (e.g., race, gender, age, diagnostic status), and key findings (e.g., racial/ethnic disparities in mental health care, barriers and facilitators for mental health service use). To ensure the accuracy of data extraction, two authors with training in quantitative research methods independently coded the reviewed studies and cross-checked for errors and disagreements. Discrepancies were addressed by re-appraisal and discussion with the first author until consensus was reached.

## Results

**Figure 1:**
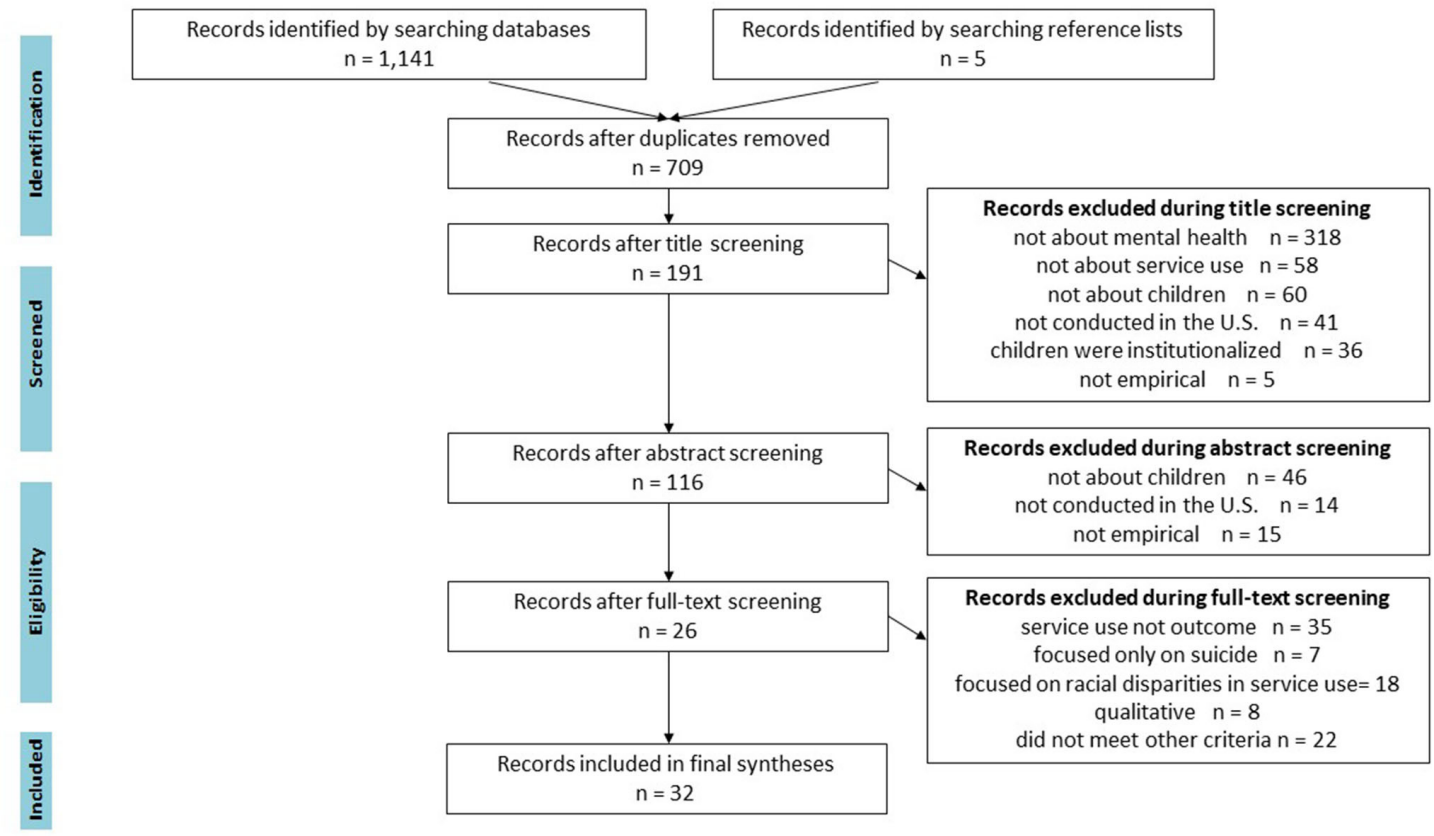
PRISMA flow diagram.

Our initial search yielded a total of 1,141 publications. After deleting duplicate publications (*n* = 436), 705 unique studies were retained for title review. As illustrated in Figure 1, 518 publications that did not meet one or more of the inclusion criteria were excluded during the title screen. After reviewing the abstracts, 75 additional studies were excluded that did not meet our inclusion criteria. A total of 116 publications passed the abstract review and were included in the full text review.

In the full text review, we excluded studies that did not have mental health service use as an outcome (*n* = 35), were purely qualitative (*n* = 8), or did not meet one or more of the other inclusion criteria (*n* = 24). Further, 25 studies with service use as the outcome were excluded because they did not examine facilitators or barriers to mental health service use. A total of 26 articles passed the full text review. Six additional studies that met all the inclusion criteria were identified by checking the reference lists of the most recently published articles. A total of 32 studies were included in the final syntheses.

### Study Characteristics

Table 1 outlines major methodological characteristics of the reviewed studies. Among the 32 studies, 26 were cross-sectional and six (18.8%) were longitudinal. Fifteen studies had sample sizes larger than 500, while 3 studies had sample sizes of smaller than 100. Over half of the studies were conducted at the community or school level, five (15.6%) analyzed administrative or electronic heath record data from one or more health agencies, five (15.6%) reported findings for nationally representative samples, and two (6.3%) were conducted at the state level.

**Table 1:** Methodological characteristics of the reviewed studies (N = 32).

**Methodological criterion**	**Description**	***n* of studies**	**(%)**
Study design	Longitudinal study	6	18.8
	Cross-sectional study	26	81.2
Sample size	Large (>500)	15	46.8
	Medium (>100 and <500)	14	43.8
	Small (<100)	3	9.4
Data collection method/data	National level	5	15.6
source	State/territory-level	2	6.3
	Community/school-level	20	62.5
	Agency administrative data	5	15.6
Data analysis	More advanced statistics	4	12.5
	Regression/analysis of covariance	27	84.4
	Bivariate statistics	1	3.1
Measurement of mental health	Reliability tested/reported	11	34.4
problem/need	Validity tested/reported	8	25
Measurement of	Reliability tested/reported	12	37.5
barriers/facilitators	Validity tested/reported	7	21.9
Theoretical framework	Reported	9	28.1
	Not reported	23	71.9

Regression was the most common method for data analysis (*n* = 27 studies, 84.4%), followed by more advanced statistical techniques (i.e., survival analysis, multilevel modeling, mixed effects modeling (*n* = 4 studies, 12.5 %). Reliability and validity of the instruments used to measure mental health problems were tested or reported in 11 (34.4%) and 8 studies (25%), respectively. Similarly, reliability and validity of the instruments used to measure factors related to mental health service use were tested or reported in 12 (37.5%) and seven (21.9%) studies, respectively.

In regard to theory utilization, nine studies (28.1%) indicated what theories informed the research, including the Anderson Behavioral Model (25), Theory of Reasoned Action (26), Aday and Anderson Access-to-Care Model (27), Pescosolido's Network-Episode Model (28), Social Determinants of Health (29), Critical Race Theory (30), Cauce et al.'s model for mental health help seeking among ethnic minority youth (31), the Behavioral Model of Health Service Use (32), and acculturation models (33, 34).

### Sample Characteristics

Table 2 summarizes main sample characteristics across the 32 reviewed studies. As to study participants, 20 of the reviewed studies (62.5%) focused exclusively on children and adolescents, two studies (6.2%) recruited only parents/guardians (predominantly mothers), eight studies (25.0%) surveyed parent-youth dyads, and two studies (6.2%) recruited multiple stakeholders (e.g., teachers and caregivers). Adolescent samples (11–20 years of age) were targeted in 12 studies, 17 studies included both children and adolescents (3–22 years of age), and 4 studies only reported median age (ranging from 12.6 to 15.3 years of age). The proportion of females in the study participants ranged from 30 to 72%, with one study sampling females exclusively (53). As to racial/ethnic composition of participants, 12 studies (37.5%) sampled mostly Blacks or African Americans, 6 studies (18.7%) focused primarily on Hispanics or Latin/a/x, including Mexican Americans and Puerto Ricans, and 4 studies (12.5%) were mostly Asian Americans (e.g., Chinese, Vietnamese). Whites were the majority participants in 10 studies (31.2%).

**Table 2:** Summary of main sample characteristics across the reviewed studies.

**Lead author, year**	**Sample characteristics**	**Mental disorders**	**Measure of mental disorders**	**Prevalence of mental disorders**	**Measure of service use**	**Prevalence of service use**	Racial/ethnic disparities in service use*
Alegria, M. 2004 (35)	1,885 caregiver-child dyads; children aged 4–18; 100% Puerto Rican	Depression, anxiety, and disruptive disorders	Computerized Diagnostic Interview for Children-IV (DISC-IV)	2.4% for depressive disorders; 11.5% for disruptive disorders: 6.5% for anxiety disorders; 22% for significant impairment	Use of school sector services and use of mental health sector services (yes/no)	School sector: 9% of males, and 5% of females; mental health sector: 8% of males, and 4% of females	N/A
Anyon, Y. 2014 (31)	8,466 high school students (9th−12th grade); 47% receiving free or reduced lunch; 20% English learners; 58% Asians; 15% Hispanics; 9% African Americans; 6% Whites; 3% Pacific Islanders; 11% multiracial	Depressive symptoms, anxiety, substance abuse	The California Healthy Kids Survey	100% diagnosed. Top three presenting issues: anxiety (27%), family issues (21%), and depression (20%)	Use of counseling and case-management services through the school-based mental health prevention initiative (yes/no)	34% of Asians; 63% of African Americans; 60% of Hispanics; 44% of Whites; 53% of Pacific Islanders; 52% of multiracial/other groups	Higher rates among African American (+); Hispanics (+); and other/ multiracial (+) groups compared to Asians Lower rates among Cambodian (–) and higher rates among other Asian groups (+), compared to Chinese
Anyon, Y. 2013 (36)	1,744 high school students (9th−12th grade); 42% Chinese Americans; 20% Latino; 9% Whites; 7% African Americans; 15% other Asian ethnic groups; 4% multiracial	Depression, suicidality, and substance abuse	Youth Risk Behavior Survey	Not reported	Use of school-based services (yes/no)	Of those who received services: 24% of Chinese; 27% of Hispanics; 20% of African Americans; 7% of Whites; 11% of other Asians; 5% of multiracial groups	Lower rates among Chinese (–) compared with Blacks
Assari, S. 2017 (37)	1,170 Black youth 13–17 years old; 69% African Americans; 31% Caribbean Blacks	Lifetime (non-psychotic) psychiatric disorders	Composite International Diagnostic Interview (CIDI)	Not reported	Medical person (yes/no)	Not reported	Lower rates among Caribbean Blacks (–) compared with African Americans
Ballard, E. 2013 (38)	442 children aged 8–18; 53% male and 47% female; 85% with public or no insurance; 21% in foster care; urban setting; 91% African Americans; 9% other race/ethnicity groups	Any psychiatric chief complaint	Emergency department intake records	100% presented with a psychiatric chief complaint to the pediatric ED; Positive suicide screen: 50% of adolescents, 48% of children	Hospitalized at index emergency department (ED) visit (yes/no) and returned to the ED for psychiatric reason (yes/no)	100% of participants used services	N/A
Bannon, W. 2008 (30)	96 parents of children aged 9- 14; mean parent age of 34; 93% mothers; 79% high school education; urban setting; 100% African Americans; 62% low-income; 95% subsidized housing	Internalizing and externalizing behaviors	Child Behavior Checklist (CBC-L)	39% of children met criteria for a clinically significant degree of externalizing behavior	CHAMP Service Use Questionnaire e.g., “Did you ever take your child for counseling?” (yes/no)	14% of parents reported using child mental health services	N/A
Burnett-Zeigler, I. 2010 (39)	85 youth-caregiver dyads; 90% female caregivers; children aged 5–18; low income; 40% female; 68% African Americans; 7% Hispanic; 2% Whites; 22% other groups	Serious emotional disturbances	Not reported	100% identified as having a serious emotional disturbance	Days of service use (school-based mental health intervention)	100% of sample used services	Lower rate in African Americans (–), compared to non-African Americans
Carson, N. 2011 (40)	252 outpatient charts of youth aged 3–20 from 5 clinical sites in a community mental health system; urban setting; 32% Haitian; 33% African Americans; 35% non-Latino Whites	ADHD, adjustment disorder, and depression	Child and Adolescent Needs and Strengths-Mental Health measure (CANSMH)	73% of Haitians, 73% of African Americans, and 63% of Whites met the definition of clinical need	Adherence to treatment (total number of sessions during the first 6 months of treatment)	100% of sample used services	Higher rate among the Whites (+), compared to Haitians
Carson, N. 2010 (29)	Medical records of 948 youth aged 3–20; urban setting; 86% Whites; 8% African Americans; 5% Haitians	ADHD; depression; anxiety	Medical records	Haitian: ADHD 33%; depression 38%; anxiety 9% African American: ADHD 32%; depression 39%; anxiety 15%	Adequate mental health treatment (receipt of at least 8 outpatient psychotherapy visits); Emergency room (ER) use (yes/no);	Receipt of adequate treatment: 43% of Whites; 39% of Haitians, 44% of African Americans ER visits: 28% of Whites;	Higher rate of ER visits among African Americans (+); less use of psychopharmacological services by Haitian and African American youth (–)
				Whites: ADHD 34%; depression 32%; anxiety 24%	Early treatment dropout (first gap in treatment for those individuals with fewer than 8 visits)	30% of Haitians; 49% of African Americans	
Erath, S. 2009 (41)	399 children aged 5–16; 51% female; 28% living with single mothers; 85% European Americans; 15% African Americans	Internalizing and externalizing behaviors	CBCL	In kindergarten: 21% showed symptoms of internalizing problems; 15% showed symptoms of externalizing problems	Use of any type of mental health service (yes/no), age of first use, and type of service	31% received services	Higher rate in Whites (+), compared to African Americans
Fisher, J. H. 2018 (42)	187 adolescents aged 12–18; 60% Hispanic; 21% African Americans; 15% Multiracial; 4% Other	Oppositional defiant disorder, conduct disorder, ADHD, mood disorder Generalized anxiety disorder, PTSD, Substance use disorder.	Adolescent-reported Youth Self-Report (YSR); caregiver reported CBCL	86% oppositional defiant disorder, 53% conduct disorder, 71%ADHD, 41% mood disorder 17% Generalized anxiety disorder, 18% PTSD, 29% Substance use disorder.	Intake attendance defined as attending an intake session at the assigned treatment site. Treatment initiation defined as the completion of an initial treatment session after completing the full intake process at the assigned treatment site.	100% were linked to services	N/A
Frosch, E. 2011 (43)	338 youth with a mean age of 12.6; 44% female; urban setting; 82% Medicaid enrolled; 80% African Americans	Mood disorder, disruptive behavior disorder, ADHD, parent-child relationship problem, anxiety disorder, adjustment disorder, substance use disorder, developmental disorder, psychosis disorder	Pediatric Psychiatry Emergency Evaluation Form (PPEEF)	100% had repeat visit to emergency department	Connection with an outpatient mental health provider (yes/no)	65% had connection with outpatient mental health provider	N/A
Goldstein, A. 2007 (44)	417 youth aged 4–18; 45% female; urban setting; 80% African Americans	Any type of mental disorders (disruptive behaviors most frequent)	Standardized written clinical assessments	100% made mental health visits to an urban pediatric emergency department	Six-month recidivism (return emergency department)	100% of sample used services	Higher rate in African Americans (+), compared to Whites
Gudino, O. G., 2008 (45)	457 youth ages 11–18; 70% males; 80% Hispanic, 20% Asian	Internalizing and externalizing behavior disorders	DISC-IV; CBCL; youth self-report	18.8% internalizing disorders; 30.9% externalizing disorders	Use of specialty and school-based mental health service use assessed prospectively over 2 years using the Services Assessment for Children and Adolescents	In general, 36.4% used specialty mental health services; 46.0% used school mental health services	Higher rates of specialty service use for Hispanics (+) compared with Asians
Gudino, O. G., 2009 (46)	1,108 youth aged 11–18; 33% female; 38.7% White; 29.3% Hispanics; 22.7% African Americans; 9.3% Asians; median household income $20,000–$24,999	Internalizing and externalizing behavior disorders	DISC-IV; CBCL; youth self-report	9% internalizing disorders; 21% externalizing disorders	Use of specialty and school-based mental health service use assessed prospectively over 2 years using the Services Assessment for Children and Adolescents	Use of specialty services: 57% of Whites; 46% of African Americans; 43% of Hispanics; 25% of Asians Use of school services: 66% of Whites; 64% of African Americans; 54% of Hispanics; 41% of Asians	Higher service rate in Whites (+) for internalizing symptoms Higher service rate in Blacks (+) for externalizing symptoms
Guo, S. 2014 (26)	1,917 students with a mean age of 12.6; 51% female; urban setting; 45% Hispanic; 49% Asians; 6% other racial/ethnic groups	Internalizing and externalizing behavior disorders	Strength and Difficulties Questionnaire (SDQ)	23% met criteria for at least one type of emotional or behavioral problem	Referral to school services (yes/no); receipt of treatment upon referral (yes/no); engagement in treatment (total number of sessions attended)	Referral to services: 20% of Asians; 73% of Hispanics	Higher referral rate in Hispanics (+), compared to Asians; No differences in receipt of treatment after referral
Guo, S. 2015 (25)	169 adolescents (10th and 11th grades); 54% female; 59% Vietnamese Americans; 41% European Americans	Emotional and behavioral symptoms	Youth self-report	Not reported	Help-seeking from potential support sources (e.g., friend, significant other, parents, teacher, mental health	Help-seeking from formal sources: 10% Europeans American; 4% Vietnamese Americans	Higher rate in European Americans (+), compared to Vietnamese Americans
					professional, doctor, and religious/spiritual leader) (yes/no)		
Ho, J. 2007 (33)	1,364 youth ages 6–17; 44% non-Hispanic White; 20% African Americans; 7% Asian/ Pacific Islander; 28% Latinos	Internalizing and externalizing behavior disorders	DISC-IV; CBCL; youth self-report	Not reported; total problem score reported instead	Any use of specialty mental health services at 2-year follow-up	Not reported	Lower rates of specialty service use in Latinos (–) and Asians (–) compared with Whites
Howell, E. 2008 (47)	65,348 youth Aged 6–17; Urban setting; 70% Whites; 15% Hispanics; 14% Black/African Americans; 1% Native Americans	Index of Mental Health Status	National Survey of America's Families	7.5% with a mental health problem	Received psychological or emotional counseling in the last year (yes/no)	8.5% Black; 17.3% Native American; 5.6% Hispanic	Rural regions: Hispanic (–) Urban regions: Hispanic (–) Black (–) Native Americans. (+)
Kim, R. 2016 (34)	93 Latino youth ages 5–15 and parent dyads	Anxiety/trauma, depression, or disruptive behavior problems	Clinical diagnosis	100% diagnosed	Session attendance, premature treatment termination, and treatment satisfaction in community-based mental health agencies	100% receiving treatment	N/A
Kodjo, C. 2004 (27)	13,570 adolescents with a mean age of 15.3 years; 50% female; 35% low-income; 67% White; 15% African Americans; 12% Hispanics	Emotional distress	Resnick's Emotional Distress Scale	29% showed symptoms	Receipt of psychological or emotional counseling in the last year (yes/no)	17% received psychological counseling in the year preceding Wave 2	Lower rate in African Americans (–) compared to Whites and Hispanics (–)
Lindsey, M. 2010 (28)	465 adolescents with a mean age of 14.8 years; 72% received free or reduced lunch; 46% female; 100% African Americans	Internalizing and externalizing behaviors	Teachers' report of whether the child need treatment or counseling	19% had internalizing problems 29% had externalizing problems	Use of any type of mental health service (yes/no) as measured by the Services Assessment for Children and Adolescents; parent report	6% currently used outpatient mental health services; 4% received school mental health services	N/A
Lindsey, M. 2010 (48)	1,621 youth aged 22 and younger (54% aged 13–17); urban setting; 43% female; 75% with public insurance; 100% African American	Mood (e.g., major depression, dysthymia, and bipolar disorder); disruptive behavior (e.g., conduct, impulsive, ADHD); and “all other diagnoses” (e.g., anxiety disorders, psychosis, other non-depressive moods)	Patient records	100% showed symptoms	Arrival status to the emergency department (involuntary vs. voluntary admission) and disposition (disposition upheld vs. dismissed)	100% of participants used services	N/A
Malhotra, K., 2015 (49)	20,970 adolescents aged 12–17; 43% female; 59% non-Hispanic White; 20% African Americans; 21% Hispanics	Externalizing behaviors	Six questions measuring the frequency of behaviors in the National Survey on Drug use and Health survey	100% displayed externalizing behaviors; Medium severity behaviors: 36.5% of Whites; 40.7% of African Americans; 38.4% of Hispanics	Use of inpatient or outpatient services (yes/no)	Mental health clinic: 5% Whites; 3% African American; 3% Hispanics Therapist: 16% Whites; 10% African American; 11% Hispanics	Lower rate of outpatient service use: African Americans (–); Hispanics (–), compared to Whites
McCabe, K. 2002 (50)	50 families with children aged 6–12; mostly low-SES; urban setting; 100% Mexican Americans; 58% of children reside in single parent family	Any type of outpatient mental health disorder	Not reported	100% of families had children admitted for any type of outpatient mental health disorders	Treatment drop-out: parents who did not return after completing the intake or one session beyond the intake	100% of participants used services	N/A
Merikangas, K. R. 2011 (13)	6,483 adolescents aged 13–18; 49% female; 65.6% non-Hispanic Whites; 15.1% non-Hispanics Blacks; 14.4% Hispanics	Mood disorders (depression, bipolar); anxiety disorders, behavior disorders (oppositional defiant disorder, conduct	Composite International Diagnostic Interview 3.0 (CIDI 3.0)	100% diagnosed	Use of any type of mental health service (yes/no) and type of service	36% of the participants received services	Lower rate of service use in Hispanics (-), compared to Whites, for severe ADHD
		disorders); ADHD; Any substance abuse (eating disorders, alcohol abuse/dependence, drug abuse/dependence)					
Mukolo, A. 2011 (51)	175 caregivers of African American youth aged 4–17	Not reported	CBCL	100% diagnosed	Caregivers reported the types settings where their child had received any behavioral health services over the past 6 months	100% of the participants received services	N/A
Williams, C. D. 2011 (52)	108 parent-adolescent dyads; Youth aged 10–17; 94% African Americans; 2% mixed races; 2% Latinos; 2% American Indians; 54% female	Internalizing and externalizing problems	DSM-IV	100% diagnosed	Number of appointments kept	100% of the participants received services	N/A
Yasui, M. 2015 (53)	1,480 girls aged 15–17; 58% African American; 42% White	Conduct disorder, major depressive disorder	Child Symptom Inventory (CSI-4)	1.9% European American girls and 4.4% African American girls diagnosed with conduct disorder; 2.6% European American girls and 3.1% African American girls diagnosed with major depressive disorder	Help-seeking from mental health professional or community service, or admittance to a hospital or in-patient clinic in the past year (yes/no)	9.6% of Whites; 11.5% of African Americans	N/A
Yeh, M. 2005 (54)	1,337 parents; Youth aged 6–17); 44.1% White; 28.8% Hispanic; 20.3% African American; 6.8% Asian and other	Internalizing and externalizing behavior disorders	DISC-IV; CBCL; youth self-report	Not reported	Any mental health services in the past year	58.4% of youths reported specialty mental health service use	Lower rates of service use at 2-year follow-up among Asians (-) and Hispanics (-) compared with Whites
Yeh, M. 2019 (55)	285 parent- youth dyads; Youth aged 12–18; 69.8% Latinx; 13.7% African American/Blacks; 5.6% Non-Hispanic White; 2.1% Asian American/ Pacific Islander; 0.4% American Indian/Alaska Native; 6.7% Multiracial	Not reported	Beliefs About Care Survey (BAC)	Not reported.	The Engagement Measure	100% of the sample received school-based or clinic-based outpatient therapy	N/A
Zerr, A. A 2014 (32)	242 parent-child dyads of children aged 4–16; 45% female; 48% Whites; 52% Hispanics	Anxiety	CBCL	100% showed symptoms	Completion of a home screen; participation in on-site diagnostic assessment (yes/no)	55% completed home screen; of those, 67% took on site diagnostic assessment	Completion of home screen: higher rate in Whites (+), compared to Hispanics

*(+) indicates statistically significant positive predictor; (–) indicates statistically significant negative predictor*.

Different instruments were used to measure mental health problems across the studies, including the Child Behavior Checklist (CBCL) (9 studies), the Diagnostic Interview Schedule for Children-IV (DISC-IV) (5 studies), the Composite International Diagnostic Interview (CIDI) (2 studies), and the Diagnostic and Statistical Manual of Mental Disorders-IV (DSM-IV) (1 study). The DISC questions elicit the diagnostic criteria specified in the DSM-IV and the WHO International Classification of Diseases, Version 10 (ICD-10). Adolescents' intake or medical records were checked in 6 studies. In another 6 studies, adolescents were asked to self-report whether they had mental health problems or needed mental health services. Other survey instruments used to assess adolescent mental health problems included the Strength and Difficulties Questionnaire (26), the Child and Adolescent Needs and Strengths-Mental Health Measure (40), the Resnick's Emotional Distress Scale (27), the Child Symptom Inventory (53), and the Beliefs About Care Survey (55). One study asked teachers to report whether the child needed treatment or counseling (28) while two studies did not report how adolescent mental health problems were assessed (39, 50).

Adolescents' diagnosis status also varied. In 13 studies (40.6%), the sample consisted entirely of youth who had been diagnosed or shown symptoms. In another 13 studies (40.6%), mental health problems were assessed in undiagnosed community-based samples of youth. The remaining 6 studies (18.2%) did not report the diagnostic status of the participants. Prevalence of mental health problems in the community samples ranged from 7.5 to 29% for any type of disorder. In general, rates were higher for externalizing problems, including ADHD and conduct disorder (15–39%) than internalizing problems, such as anxiety and depression (9–21%).

Most studies defined service use as having made contact or had a visit with a mental health professional (e.g., psychiatrist, school counselor, emergency department). Yet, specific types of service use varied across the reviewed studies. In 14 studies (43.7%), researchers examined the use of any type of mental health service, such as specialty, inpatient, outpatient, or school-based services, or any combination of services. Fourteen studies (43.7%) investigated specialty/clinic-based or outpatient mental health service use, six studies (18.7%) examined the use of school-based mental health services, and three studies (9.37%) focused on the use of services in emergency department or hospital settings. Further, 11 out of the 32 studies (34.6%) examined the duration or intensity of service use, including treatment drop-out and unmet need, and only one study (48) examined admittance outcomes (i.e., whether admittance was for a psychiatric or non-psychiatric reason and whether admittance was a voluntary or involuntary).

### Racial/Ethnic Disparities in Youth Mental Health Service Use

As further listed in Table 2, 30 studies (93.7%) reported rates of service use, ranging from 3 to 100%, among which 15 studies identified racial/ethnic disparities in the rate of mental health service use. In general, compared with Whites, significantly lower levels of service use were observed among racial/ethnic minorities. However, patterns of racial/ethnic disparities in service use varied by service type.

Specifically, compared with Whites, Blacks and African Americans were less likely to use any type of mental health service (13, 41, 47), specialty or outpatient services (e.g., therapy, counseling, psychopharmacological services) (29, 46, 47, 49), and school-based services (39, 46). Yet, as identified in two studies (29, 44), the likelihood that African Americans used emergency department services was much higher than that of Whites. One study examined within-group differences between African Americans and Haitians and reported similar odds in their receipt of treatment for ADHD and depression; however, Haitians were less likely to remain in treatment than African Americans and Whites if they did not meet a standard of clinical need (40).

Similarly, compared with Whites, Latinos and Hispanics were generally less likely to use any type of service (13, 32) and specialty or outpatient services (33, 47, 49). In two studies, Asian Americans were found less likely to use any type of mental health service (25) and specialty services (33) than White adolescents. Further, compared with Blacks and Latinos/Hispanics, Asian Americans were less likely to use or be referred to school-based services (26, 31, 35) and among Asian American subgroups, Cambodian youth were particularly less likely to receive school-based mental health services (31).

### Barriers and Facilitators for Mental Health Service Use Among Minority Youth

Extensive factors influencing mental health service use among minority adolescents were identified across the 32 studies. Based on the Social Ecological Model, we categorized the identified barriers and facilitators at five different levels, i.e., adolescent, parent/family, service provider, contextual/structural, and social/cultural levels.

### Adolescent-Related Factors

As listed in Table 3, 21 studies identified adolescent-related barriers and facilitators of mental health service use, including biological (e.g., age, gender), clinical (e.g., symptom severity), behavioral (e.g., drug/alcohol use), and psychological characteristics (e.g., internal asset) of minority youth. Specifically, in 3 studies, minority adolescents' disorder types (i.e., internalizing or externalizing problems) were found to negatively influence their use of mental health services (41, 46, 48). Gender was identified as a barrier in 3 studies, with conflicting findings being reported (31, 35, 55). In two studies, adolescent perceived barriers to care (e.g., not wanting parents to know, being afraid of what the doctor would say) (27) and self-perceived stigma (e.g., perceived service use as being for troublemakers, feeling of comfort) (36) were noted as barriers. Other barriers included youth's ethnic identity (i.e., an individual's affirmation of and belonging to an ethnic group) (53) and older age (45).

**Table 3:** Categorization of barriers and facilitators^*^ for mental health service use among racial/ethnic minority youth by the Social Ecological Model (*N* = 32 studies).

**Adolescent-related factors(*n* = 21 studies)**	**Parent-related factors (*n* = 10 studies)**	**Therapeutic/service provider-related factors (*n* = 9 studies)**	**Contextual/structural factors (*n* = 15 studies)**	**Social/cultural factors (*n* = 9 studies)**
**Barriers:** Disorder type: Externalizing or behavioral problems (48)Internalizing problems (41, 46)Gender: Female (35)Male (31, 55)Adolescent self-perceived barriers or stigma to care (27, 36) Ethnic identity (53) Older age (45)	**Barriers:** Parent perceived barriers and fear (50, 51) Parenting issues: Parent reliance on discipline (50) Caregiver attachment (53) Lower education level (39, 50) Older age (39)	**Barriers:** N/A	**Barriers:** Income: Low income for adequate treatment (29); Low income for emergency services (48); Low income in urban region (47); Low income for inpatient services (49) Insurance type: Medicaid for adequate treatment (29); Having no insurance (29, 47); Having other insurance (47) Family structure: Two-parent household (31); No. of female headed household in the neighborhood (29) Geographic location: Blacks and Hispanics in urban region (47); Blacks in rural region (13); Hispanics in rural region (47); Living in urban Midwest region (47); Living in rural Midwest or south region (47) % of Asians in school (31)	**Barriers:** Acculturation: High acculturation families (34); High parental affinity to an alternative culture among Latinos and Asian Americans (33); Mid-range cultural pride reinforcement of parents (30)Negative social relationship (36) Parent perceived racial discrimination/prejudice (54)
**Facilitators:** Diagnosis status/symptom severity (13, 25, 26, 28, 31–33, 35, 37, 40, 44, 47, 48, 53, 54) Disorder type: Externalizing problems/ disorder: (26, 28, 31, 35, 41, 45, 46, 48, 53)Internalizing problems/disorders (28, 45)Gender: Female (25, 31, 39–42, 45)Male (13, 28, 47)Suicidality (27, 38, 48) Older age (40, 42, 47) Internal assets (31) Poor self-rated health (37) Being in foster care (38)	**Facilitators:** Parent perceived need and etiologic beliefs: Parent-youth concordance on perceived need for mental health services (52); Not parent's idea for treatment (39); Parental concern about the child's behavior, emotional state, or ability to learn (35); Parent beliefs of physical causes, relational issues, and trauma (54) Family stressors or parental strains: Caregiver strain (39); Family stressful life events (25); Parental hassles (30) Parent health: Poor parental health (47); Parents without health problems (39) Extended family caring (30) Female gender (39)	**Facilitators:** Patient/clinician ethnic match (50, 51) Positive relationship or connection with health providers (36, 43) Therapeutic attributes (e.g., inpatient disposition after visit, arrival at emergency services) (43, 48) Prior receipt of intervention/services/ hospitalization (28, 44) Youth-therapist co-endorsement of etiological beliefs (55) Professional referral by primary care providers, pediatricians, school psychologists, or counselors for home screening (32)	**Facilitators:** Insurance: Having health insurance (45); Public insurance (e.g., Medicaid) for emergency services (29, 38) or counseling (47) Private insurance for school-based services (39) or emergency services (48) No insurance for emergency services (38, 40) Higher household income (33, 42, 54, 55) Low SES: Low income for school-based services (39); Parent unemployment for school-based services (39); Worse subjective socioeconomic status (37) Family structure: single parent household (13, 47, 49) Blacks and Whites living in urban area (27) Enabling resources such as family/work schedule, availability of childcare, transportation (32)	**Facilitators:** Difficulty or negative school experiences (Racial discrimination by school adults and peers, Lower sense of agency in school, being expelled or suspended, etc.) (25, 26, 31, 35, 38) Adolescent perceived social support network (28) School assets (31) Teacher referrals or teacher reported need for service (28, 36) Mid-range spiritual/religious coping (30)

* *p < 0.05*.

The most frequent adolescent-related facilitator of mental health service use was minority youth's diagnosis status/severity of symptoms. As reported in 15 studies, youth who were diagnosed with a mental health problem and/or had severe impairment were more likely to use services than those who did not show symptoms (13, 25, 26, 28, 31–33, 35, 37, 40, 44, 47, 48, 53, 54). In 10 studies, disorder types were identified as facilitators of minority adolescents' mental health service use, including externalizing problems (26, 28, 31, 35, 41, 45, 46, 48, 53) and internalizing problems (28, 45). Being female (25, 31, 39–42, 45), being male (13, 28, 47), or having suicidality (27, 38, 48) further increased adolescents' likelihood of using services. As identified in 3 studies (40, 42, 47), older minority adolescents were more likely to use mental health services. In one study, minority youth were more likely to use school-based services when they had more internal assets, such as self-efficacy, empathy, self-awareness, and goals and aspirations (31). Additional adolescent-level facilitators of mental health service use included having poor self-rated health (37) and being in foster care (38).

### Parent-Related Factors

Ten studies identified parent-related factors that influenced minority adolescent mental health service use, including parental perceptions and beliefs, family and parenting issues, and demographic characteristics. Specifically, three studies identified parent perceptions and stigmatized attitudes as significant barriers to adolescent use of mental health services, including parents' self-perceived barriers to treatment and fear of child being labeled by professionals as having a mental health disorder (50). As to parenting issues, adolescents with parents who relied heavily on discipline (50) and who fostered strong caregiver attachment (53) were found to be less likely to use mental health services than their counterparts. Demographic barriers included older ages (39) and lower educational level of parents (39, 50).

Parents' perceptions and beliefs that play facilitating roles in minority adolescents' mental health service use included parent-youth concordance on perceived need for mental health services (52), parental concern about the child's behavior, emotional state, or ability to learn (35) and parental beliefs of physical causes, relational issues, and trauma among Asians and Hispanics (54). Yet, in one study with predominantly African American participants (39), adolescents of parents who said it was not their idea to get treatment for their child received more days of service in a school-based mental health intervention program. Further, three studies identified family stressors or parental strains as facilitators for adolescent mental health service use, including parents' perceptions about daily situations that may act as stressors for them (25, 30, 39). Parental health conditions were noted as facilitators in two studies, with conflicting findings being reported (39, 47). Lastly, in one study with African American parents in urban settings, adolescents with extended family caring were more likely have received mental health services (39).

### Therapeutic/Service Provider-Related Factors

No statistically significant barriers to mental health service use were reported at the therapeutic/service provider level, whereas nine studies identified various facilitators. In two studies, researchers found ethnic match between patient and practitioner to be a facilitator (50, 51); minority youth were more likely to use mental health services when their mental health practitioners were of the same racial/ethnic group. Another two studies identified prior participation in intervention (28) or receipt of mental health services/hospitalization as a significant facilitator (44). Therapeutic attributes were further noted as indicators of service use, including time of day youth arrived at emergency services (48) and inpatient disposition after visit (43).

Two studies reported relationship with providers as a facilitator for mental health service use. Specifically, Anyon et al. (36) found that having positive relationships with school health professionals encouraged adolescents' use of school-based mental health services; similarly, Frosch (43) noted that connection to outpatient providers facilitated service use among minority children. Further, one study with a majority Latinx population reported that youth-therapist co-endorsement of etiological beliefs as a facilitator of mental health service use (55). Lastly, professional referral was identified as a facilitator in one study (32).

### Contextual/Structural Factors

Fifteen studies identified factors influencing minority adolescent mental health service use at the contextual/structural level, including household income, insurance status, and family structure. As noted in four studies, low household income significantly decreased adolescents' likelihood of using adequate treatment overall (29, 47), emergency services (48), and inpatient services (49). Insurance status was the second common barrier. Compared with those covered by private insurance, minority adolescents with Medicaid (29), having no insurance (29, 47), or having other insurance (47) were much less likely to use mental health services. Findings regarding family structure were inconsistent. One study with predominantly Asian and Latino/a youth found that youth from two-parent households were less likely to use school-based mental health services than those from single-parent households (31); in another study, African American youth from neighborhoods with a high proportion of female headed households were found to be less likely to use specialty mental health services than youth from male or dual headed households (29). Region of residence and study setting (rural vs. urban) were further found to influence minority adolescent mental health service use in two studies, but inconsistent findings were reported (13, 47).

As to facilitators at the contextual/structural level, insurance status was the most reported factor. As noted in six studies, having insurance—regardless of specific types (i.e., public or private insurance)—significantly increased adolescents' likelihood of using emergency services (29, 38, 48), school-based services (39), counseling (47), or any type of service (45). Yet, two studies found that minority adolescents with no insurance were more likely to use emergency services (38, 40). Similarly, although four studies identified higher household income as a facilitator of minority adolescent mental health service use (33, 42, 54, 55), one study reported that adolescents from low-income households and those with an unemployed parent were more likely to use school-based mental health services (39). Another study further identified worse subjective socioeconomic status as a positive predictor among African American and Caribbean Black youth (37). Living in single-parent households significantly increased the likelihood that minority adolescents used services, as noted in three studies (13, 47, 49). Other facilitators included living in urban setting (27) and having enabling resources such as family/work schedule, availability of childcare, transportation (32).

### Social/Cultural Factors

Acculturation acted as a barrier to service use among minority youth; however, mixed findings were observed. In one study, for example, Kim et al. (34) found Latino/a children with less acculturated caregivers were more likely to terminate mental health service whereas those with highly acculturated caregivers were more likely to not show up for services. In contrast, Ho et al. (33) noted that high parental affinity to another culture was a negative predictor of service use among Latino/a and Asian youth. In another study with an urban sample of African American families, children of parents with moderate cultural pride reinforcement were least likely to have used mental health services (30). Other barriers included negative social relationship among minority high school students (36) and parent perceived racial discrimination/prejudice (54).

Most of the social facilitators center around adolescent school experiences. As noted in five studies, minority adolescents were more likely to use mental health services across settings when they had difficulties or negative experiences in school, including racial discrimination by school adults and peers, low sense of agency in school, or being expelled or suspended (25, 26, 31, 35, 38). In contrast, two studies reported that when adolescents felt socially supported (e.g., teachers reported a need for services) (28) or perceived school assets (31), they were more likely to use school-based mental health services. Teacher referral played a major role in promoting minority adolescents' use of mental health services, as observed in two studies (28, 36). Lastly, one study with African American families found that adolescents of parents with moderate spiritual or religious coping were most likely to have used mental health services (30).

## Discussion

This systematic review synthesizes recent evidence on barriers and facilitators of mental health service use among racial/ethnic minority adolescents in the U.S. A detailed appraisal of findings from 32 quantitative studies highlight critical research gaps in the literature and suggest key areas of potential intervention to promote mental health service use among minority adolescents.

In this review, we have identified a plethora of barriers and facilitators at different socioecological levels that can influence minority adolescents' access, attendance, and adherence to mental health treatment. At the individual level, consistent with research in White adolescents, minority adolescents' diagnosis status, levels of symptom severity, and disorder types are major facilitators for their mental health service use. Compared with internalizing problems, externalizing problems tend to negatively impact the lives of people around adolescents and, consequently, are more readily identified by caregivers. In contrast, the symptoms of internalizing problems may be more difficult for caregivers to detect (45). As such, adolescents with internalizing problems are usually at greater risk of having their mental health needs unmet. Community-based efforts are needed, therefore, to raise minority parents' awareness of adolescent mental health issues. Notably, several studies identified adolescent perceptions, attitudes, and ethnic identity-related cultural stigma as salient barriers to mental health service use while internal assets as facilitators. School-based psychoeducational campaigns, therefore, should target normalizing mental health service use and promoting help-seeking self-efficacy in minority adolescents.

Not surprisingly, most barriers and facilitators at the parent level surround parents' attitudes, etiologic beliefs, perceptions of service needs, and concerns and fears. Adolescents with mental disorders generally do not seek mental health services themselves. Rather, it is adult gatekeepers, often parents, who seek out services on behalf of them. In fact, research has suggested that between 40 and 55% of 15- to 17-year-old adolescents identify family as the major influence on their mental health help-seeking behavior (56). Community outreach programs, therefore, should focus on promoting mental health literacy and de-stigmatizing mental health service use among minority parents. Parenting issues and parental stress are other salient factors that can influence minority adolescents' service use. Racial/ethnic minority parents, especially new immigrants, are often at high risk for behavioral and emotional problems due to various immigration-related financial, social, and cultural stressors. Such parental distress, if handled improperly, can lead to disrupted family dynamics and thereby unrecognized mental health service needs among minority adolescents. Parenting programs should consider incorporating stress management and emotion regulation skills for parents so as to build family resilience and promote mental health among immigrant parents and adolescents.

Unexpectedly, at the treatment or service provider level, the studies that we reviewed did not report any statistically significant barriers to service use, which highlights the need for more research with clinical adolescent samples to decipher the idiosyncrasies of their help-seeking process. Most facilitators that we identified are related to therapist characteristics (e.g., client and therapist ethnic match), therapeutic interaction (e.g., client and therapist co-endorsement of etiological beliefs), and treatment process and delivery (e.g., referral sources and disposition after visit). Ethnic match between client and therapist is a critical way to improve the cultural sensitivity of services, which in turn can help increase treatment attendance and retention among minority adolescents and parents (50). Further, discrepancies between clients and healthcare providers in etiological beliefs may lead to differential expectations regarding treatment goals and approaches that may affect treatment compliance and outcomes. Taken together, these findings pinpoint the importance of establishing effective communication and building therapeutic relationship between therapists, adolescents, and parents to ensure treatment engagement. Specialty mental health clinics should also strengthen their relationship and service coordination with other healthcare providers (e.g., primary care physicians, pediatricians, school counselors) to facilitate client referral.

At the contextual and structural level, low household income and lack of insurance were key barriers to mental health service use among minority adolescents. This is not surprising and underscores the importance of providing necessary social support to low-income minority families. In 2010, the Affordable Care Act expanded Medicaid eligibility to cover more low-income families and, since then, has benefited millions of children and their families nationwide (7). To make sure adolescents in low-income families receive needed care, more efforts are warranted to enroll eligible adolescents in Medicaid and to provide safety-net mental health services to adolescents who are ineligible for Medicaid. More research attention and social support are also needed for minority adolescents from single-parent families, who have been identified as having higher rates of service needs (6). Notably, one study identified enabling resources such as transportation, family/work schedule, and availability of childcare as salient facilitators. Future mental health treatments should incorporate such accommodations in their program planning and consider using the emerging e-mental health strategies to deliver services to immigrant parents and adolescents with busy work and school schedules.

As identified in this review, the role that acculturation plays in promoting or prohibiting minority adolescents' mental health service use is inconsistent across studies. In general, due to stronger adherence to heritage cultural attitudes about mental illness and stigma and less familiarity with social problems affecting adolescents in the host culture, foreign-born immigrant parents may be less likely than native-born minority parents to seek out mental health services for their children. In order to deliver treatment in a culturally congruent manner, therapists and healthcare providers are encouraged to discuss cultural issues with parents. Consistent with ecological theories of help-seeking among ethnic minority youth that emphasize organizational and social influences on adolescents' pathways into services, this review highlights teacher referral, school assets, and perceived social support network in facilitating minority adolescents' mental health service use. Importantly, emerging research suggests that cultural stereotype can disguise problem recognition among key gatekeeps for minority adolescents. For example, teachers often expect Asian students to be quiet, anxious, and perfectionistic (57). Such cultural stereotypes can lead to overlooked depressive symptoms among Asian students. Psychoeducation in schools is important, therefore, to raise teachers' awareness of cultural variations in the exhibition of symptoms of mental disorders. Community outreach programs in disadvantaged racial/ethnic communities are also encouraged to build strong social network support and facilitate problem recognition in minority populations.

Several critical research gaps in the literature emerged from this systematic review. First, the small number of race/ethnicity-specific studies suggest that little attention has been paid to the mental health service needs of adolescents of certain racial/ethnic groups, e.g., Asian Americans. There is even less attempt to clarify any within-group differences between racial/ethnic subgroups. Blacks, Hispanics and Asian Americans all include hugely heterogeneous populations with distinctive cultural norms and values that may lead to differential patterns of mental health service need and use (58). Without knowledge of culture-specific factors influencing the help-seeking process of adolescents in these racial/ethnic subgroups, effective interventions to engage them into treatment are not feasible. To make sure adolescents receive mental health services that they need, more ethnicity-specific research and culturally tailored treatment engagement interventions are necessary.

Second, more setting-specific research is needed to investigate factors influencing minority adolescents' service use in school, hospital, and multiple settings. Adolescents spend most of their daily life in school; as a result, schools are often one of the first places where adolescents' mental health needs are recognized and initially addressed (8, 59). Nevertheless, we identified only six studies that examined school-based service use among minority adolescents. To make sure minority adolescents receive timely services, better understanding of factors influencing their service use in school settings is necessary. Likewise, more attention is needed to clarify minority adolescents' pathways to emergency services or service use in general medical settings. Based on two studies identified in this review (29, 60), minority adolescents, especially Blacks, are more likely to use emergency services than Whites. The higher representation of minority adolescents in emergency settings may be due to inadequate access to outpatient mental health care (29). To make sure minority adolescents do not delay needed care until symptoms become urgent, we need more research to understand their use of emergency or inpatient mental health services. Moreover, although a couple of studies have investigated minority adolescents' multi-setting service use, there was no inquiry about why or how linkages were made across settings. To inform better cross-setting partnership, future work is needed to clarify the processes that influence adolescent multi-setting mental health service use.

Furthermore, noticeably absent in the reviewed studies are the perspectives of healthcare providers who are at the frontline of serving minority adolescents and families. Previous research has shown that ethnic, gender, and language matches between clients and counselors often result in better outcome for racial/ethnic minorities (61). To engage minority adolescents and families into mental health treatment and make sure they receive culturally responsive services, we need better understanding of the challenges that mental healthcare professionals' face when serving minority adolescents and families, as well as the strategies they use during the therapeutic process. Additionally, 87% of U.S. adolescents have a primary care physician, who are often considered as “gatekeepers” to adolescents' mental health services (62–64). Through routine checkups, physicians are at the frontline of recognizing mental health concerns in adolescents (65, 66). It is important, therefore, that we understand primary care physicians' experiences working with racial/ethnic minority families and their perceptions of barriers and facilitators for effective management of minority adolescents' mental health problems.

There are multiple methodological limitations and theoretical issues in the literature that had implications for the current review. First, most of the studies we identified were cross-sectional in design, which made it difficult to derive causal relationships between identified barriers and facilitators and the service use outcome. Six studies were longitudinal, but half of them seemed to be from the same research project. While producing multiple articles from a well-designed project is common, the repeated findings, shared methodological limitations, and overlapping samples complicated interpretation of our findings. Second, most studies neglected reliability and validity testing for tools they used to measure mental health problems and barriers and facilitators for service use. Such neglect can lead to measurement errors and affect interpretation of the results. This is especially problematic for healthcare disparities research given the huge social, contextual, and cultural variations among different racial/ethnic groups that may influence their health behaviors. Further, the level of theory utilization among the reviewed studies was low. Over 70% of the studies were not theoretically driven or used theories superficially. Theories provide a framework for identifying determinants of health behaviors such as mental health service use. To facilitate the development of culturally tailored interventions to engage racial/ethnic minority adolescents into treatment, more conceptually grounded predictors of mental health service use are needed in future exploratory studies.

This review also has several limitations. First, as mentioned above, because of the small number of studies that focused on some racial/ethnic minority groups (e.g., Asian Americans), we were not able to carry out more detailed race/ethnicity-specific analyses, which precludes many conclusions being drawn. Second, in our search strategy, we used only seven databases to search for studies that met our inclusion criteria. Thus, articles published in journals not indexed by these databases may have been missed for inclusion. Third, we were not able to do a meta-analysis of the studies due to methodological heterogeneity among the included quantitative studies. Finally, the review process for inclusion of studies that meet our inclusion criteria is subjective, as it is for all systematic review studies. Despite these limitations, this study is the first systematic review that summarizes and assesses existing evidence of barriers and facilitators of mental health service use among racial/ethnic minority adolescents in the U.S. Findings from this study hold direct implications for future research and treatment engagement interventions.

## Data Availability Statement

The original contributions generated for the study are included in the article, further inquiries can be directed to the corresponding author/s.

## Author Contributions

WL conceptualized the research question. WL, AT-R, and MM conducted literature review, synthesized findings, and drafted the manuscript. MM-L, AY, and LX participated in drafting and revising the manuscript. All authors approved the final version of this manuscript.

## Conflict of Interest

The authors declare that the research was conducted in the absence of any commercial or financial relationships that could be construed as a potential conflict of interest.

## References

[1] American Psychiatric Association (2013). Diagnostic and Statistical Manual of Mental Disorders. 5th ed. Arlington, VA.

[2] Coghill D Sonuga-Barke EJS. Annual research review: categories versus dimensions in the classification and conceptualisation of child and adolescent mental disorders-implications of recent empirical study. J Child Psychol Psychiatry. (2012) 53:469–89. 10.1111/j.1469-7610.2011.02511.x 22288576

[3] Steinberg L Morris AS. Adolescent development. Annu Rev Psychol. (2001) 52:83–110. 10.1146/annurev.psych.52.1.83 11148300

[4] Kessler RC Angermeyer M Anthony JC De Graaf RO Demyttenaere K Gasquet I. Lifetime prevalence and age-of-onset distributions of mental disorders in the World Health Organization's World Mental Health Survey Initiative. World Psychiatry. (2007) 6:168. 10.1017/s2045796015000566 18188442 PMC2174588

[5] Merikangas KR He JP Burstein M Swanson SA Avenevoli S Cui L. Lifetime prevalence of mental disorders in US adolescents: results from the National Comorbidity Survey Replication–Adolescent Supplement (NCS-A). J Am Acad Child Adolesc Psychiatry. (2010) 49:980–9. 10.1016/j.jaac.2010.05.017 20855043 PMC2946114

[6] Lu W. Child and adolescent mental disorders and health care disparities: results from the National Survey of Children's health, 2011–2012. J Health Care Poor Underserved. (2017) 28:988–1011. 10.1353/hpu.2017.0092 28804073

[7] Lu W. Adolescent depression: national trends, risk factors, and healthcare disparities. Am J Health Behav. (2019) 43:181–94. 10.5993/AJHB.43.1.15 30522576

[8] Lu W. Treatment for adolescent depression: national patterns, temporal trends, and factors related to service use across settings. J Adolesc Health. (2020) 67:401–8. 10.1016/j.jadohealth.2020.02.019 32331929

[9] Auerbach RP Kim JC Chango JM Spiro WJ Cha C Gold J. Adolescent nonsuicidal self-injury: examining the role of child abuse, comorbidity, and disinhibition. Psychiatry Res. (2014) 220:579–84. 10.1016/j.psychres.2014.07.027 25095754 PMC4252370

[10] Naicker K Galambos NL Zeng Y Senthilselvan A Colman I. Social, demographic, and health outcomes in the 10 years following adolescent depression. J Adolesc Health. (2013) 52:533–8. 10.1016/j.jadohealth.2012.12.016 23499382

[11] Centers for Disease Control and Prevention. Leading Causes of Death Reports, 1981–2015. WISQARS (2017). Available online at: https://webappa. cdc. gov/sasweb/ncipc/leadcause.html (accessed December 19, 2017).

[12] Planey AM Smith SM Moore S Walker TD. Barriers and facilitators to mental health help-seeking among African American youth and their families: a systematic review study. Child Youth Serv Rev. (2019) 101:190–200. 10.1016/j.childyouth.2019.04.001

[13] Merikangas KR He JP Burstein M Swendsen J Avenevoli S Case B. Service utilization for lifetime mental disorders in US adolescents: results of the National Comorbidity Survey–Adolescent Supplement (NCS-A). J Am Acad Child Adolesc Psychiatry. (2011) 50:32–45. 10.1016/j.jaac.2010.10.006 21156268 PMC4408275

[14] Reardon T Harvey K Baranowska M O'Brien D Smith L Creswell C. What do parents perceive are the barriers and facilitators to accessing psychological treatment for mental health problems in children and adolescents? A systematic review of qualitative and quantitative studies. Eur Child Adolesc Psychiatry. (2017) 26:623–47. 10.1007/s00787-016-0930-6 28054223 PMC5446558

[15] Velasco AA Santa Cruz IS Billings J Jimenez M Rowe S. What are the barriers, facilitators and interventions targeting help-seeking behaviours for common mental health problems in adolescents? A systematic review. BMC Psychiatry. (2020) 20:1–22. 10.1186/s12888-020-02659-0 32527236 PMC7291482

[16] O'Brien D Harvey K Howse J Reardon T Creswell C. Barriers to managing child and adolescent mental health problems: a systematic review of primary care practitioners' perceptions. Br J Gen Pract. (2016) 66:e693–707. 10.3399/bjgp16X687061 27621291 PMC5033306

[17] Moher D Liberati A Tetzlaff J Altman DG. Preferred reporting items for systematic reviews and meta-analyses: the PRISMA statement. Int J Surg. (2010) 8:336–41. 10.1016/j.ijsu.2010.02.007 20171303

[18] World Health Organization. Adolescent Friendly Health Services: An Agenda for Change. Geneva: World Health Organization (2003).

[19] Sawyer SM Azzopardi PS Wickremarathne D Patton GC. The age of adolescence. Lancet Child Adolesc Health. (2018) 2:223–8. 10.1016/S2352-4642(18)30022-1 30169257

[20] Stein DJ Phillips KA Bolton D Fulford KW Sadler JZ Kendler KS. What is a mental/psychiatric disorder? From DSM-IV to DSM-V. Psychol Med. (2010) 40:1759–65. 10.1017/S0033291709992261 20624327 PMC3101504

[21] Green CA Estroff SE Yarborough BJ Spofford M Solloway MR Kitson RS. Directions for future patient-centered and comparative effectiveness research for people with serious mental illness in a learning mental health care system. Schizophr Bull. (2014) 40:S1–94. 10.1093/schbul/sbt170 24489078 PMC3911266

[22] Garrard J. Health Sciences Literature Review Made Easy: the Matrix Method. Burlington, MA: Jones & Bartlett Learning (2017).

[23] Lu W McKyer EL Lee C Goodson P Ory MG Wang S. Perceived barriers to children's active commuting to school: a systematic review of empirical, methodological and theoretical evidence. Int J Behav Nutr Phys Act. (2014) 11:140. 10.1186/s12966-014-0140-x 25403958 PMC4245777

[24] Lu W Diep CS McKyer EL. Risk factors for childhood obesity among Asian Americans: a systematic review of literature and recommendations for health care research. J Health Care Poor Underserved. (2015) 26:171–90. 10.1353/hpu.2015.0056 25981097

[25] Guo S Nguyen H Weiss B Ngo VK Lau AS. Linkages between mental health need and help-seeking behavior among adolescents: moderating role of ethnicity and cultural values. J Couns Psychol. (2015) 62:682. 10.1037/cou0000094 26376178 PMC4605858

[26] Guo S Kataoka SH Bear L Lau AS. Differences in school-based referrals for mental health care: understanding racial/ethnic disparities between Asian American and Latino youth. School Ment Health. (2014) 6:27–39. 10.1007/s12310-013-9108-2

[27] Kodjo CM Auinger P. Predictors for emotionally distressed adolescents to receive mental health care. J Adolesc Health. (2004) 35:368–73. 10.1016/S1054-139X(04)00061-8 15488430

[28] Lindsey MA Barksdale CL Lambert SF Ialongo NS. Social network influences on service use among urban, African American youth with mental health problems. J Adolesc Health. (2010) 47:367–73. 10.1016/j.jadohealth.2010.01.025 20864006 PMC2945602

[29] Carson N Cook B Alegría M. Social determinants of mental health treatment among Haitian, African American and white youth in community health centers. J Health Care Poor Underserved. (2010) 21 (2 Suppl.):32. 10.1353/hpu.0.0297 20453375 PMC3190591

[30] Bannon Jr WM Cavaleri MA Rodriguez J McKay MM. The effect of racial socialization on urban African American use of child mental health services. Soc Work Ment Health. (2008) 6:9–29. 10.1080/15332980802032326 20228964 PMC2836727

[31] Anyon Y Ong SL Whitaker K. School-based mental health prevention for Asian American adolescents: risk behaviors, protective factors, and service use. Asian Am J Psychol. (2014) 5:134. 10.1037/a0035300

[32] Zerr AA Pina AA. Predictors of initial engagement in child anxiety mental health specialty services. Child Youth Care forum. (2014) 43:151–64. 10.1007/s10566-013-9230-1 24683301 PMC3964616

[33] Ho J Yeh M McCabe K Hough RL. Parental cultural affiliation and youth mental health service use. J Youth Adolesc. (2007) 36:529–42. 10.1007/s10964-006-9114-x

[34] Kim RE Lau AS Chorpita BF. The impact of Latino caregiver acculturation on treatment engagement in children's community mental health services. J Child Family Stud. (2016) 25:891–901. 10.1007/s10826-015-0259-7

[35] Alegría M Canino G Lai S Ramirez RR Chavez L Rusch D. Understanding caregivers' help-seeking for Latino children's mental health care use. Med Care. (2004) 42:447–55. 10.1097/01.mlr.0000124248.64190.56 15083105

[36] Anyon Y Whitaker K Shields JP Franks H. Help-seeking in the school context: understanding Chinese American Adolescents' Underutilization of School Health Services. J School Health. (2013) 83:562–72. 10.1111/josh.12066 23834608

[37] Assari S Caldwell CH. Mental health service utilization among black youth; psychosocial determinants in a national sample. Children. (2017) 4:40. 10.3390/children4050040 28513567 PMC5447998

[38] Ballard ED Horowitz LM Jobes DA Wagner BM Teach SJ. Association of positive responses to suicide screening questions with hospital admission and repeat emergency department visits in children and adolescents. Pediatr Emerg Care. (2013) 29:1070–4. 10.1097/PEC.0b013e3182a5cba6 24076609 PMC3819122

[39] Burnett-Zeigler I Lyons JS. Caregiver factors predicting service utilization among youth participating in a school-based mental health intervention. J Child Family Stud. (2010) 19:572–8. 10.1007/s10826-009-9331-5

[40] Carson NJ Stewart M Lin JY Alegria M. Use and quality of mental health services for Haitian youth. Ethn Health. (2011) 16:567–82. 10.1080/13557858.2011.586024 22050537 PMC3226766

[41] Erath SA Keiley MK Pettit GS Lansford JE Dodge KA Bates JE. Behavioral predictors of mental health service utilization in childhood through adolescence. J Dev Behav Pediatr. (2009) 30:481. 10.1097/DBP.0b013e3181c35938 19890217 PMC2810267

[42] Fisher JH Lichvar E Hogue A Dauber S. Perceived need for treatment and engagement in mental health services among community-referred racial/ethnic minority adolescents. Adm Policy Ment Health Ment Health Serv Res. (2018) 45:751–64. 10.1007/s10488-018-0863-0 29525929 PMC6064387

[43] Frosch E DosReis S Maloney K. Connections to outpatient mental health care of youths with repeat emergency department visits for psychiatric crises. Psychiatr Serv. (2011) 62:646–9. 10.1176/ps.62.6.pss6206_0646 21632734

[44] Goldstein AB Frosch E Davarya S Leaf PJ. Factors associated with a six-month return to emergency services among child and adolescent psychiatric patients. Psychiatr Serv. (2007) 58:1489–92. 10.1176/ps.2007.58.11.1489 17978263

[45] Gudino OG Lau AS Hough RL. Immigrant status, mental health need, and mental health service utilization among high-risk Hispanic and Asian Pacific Islander youth. Child Youth Care Forum. (2008) 37:139. 10.1007/s10566-008-9056-4

[46] Gudiño OG Lau AS Yeh M McCabe KM Hough RL. Understanding racial/ethnic disparities in youth mental health services: do disparities vary by problem type?. J Emot BehavDisord. (2009) 17:3–16. 10.1177/106342660831771031192675

[47] Howell E McFeeters J. Children's mental health care: differences by race/ethnicity in urban/rural areas. J Health Care Poor Underserved. (2008) 19:237–47. 10.1353/hpu.2008.0008 18263999

[48] Lindsey MA Joe S Muroff J Ford BE. Social and clinical factors associated with psychiatric emergency service use and civil commitment among African-American youth. Gen Hosp Psychiatry. (2010) 32:300–9. 10.1016/j.genhosppsych.2010.01.007 20430234 PMC2862230

[49] Malhotra K Shim R Baltrus P Heiman HJ Adekeye O Rust G. Racial/ethnic disparities in mental health service utilization among youth participating in negative externalizing behaviors. Ethn Dis. (2015) 25:123–9. 10.1007/s40615-018-00536-x 26118137

[50] McCabe KM. Factors that predict premature termination among Mexican-American children in outpatient psychotherapy. J Child Family Stud. (2002) 11:347–59. 10.1023/A:1016876224388

[51] Mukolo A Heflinger CA. Rurality and African American perspectives on children's mental health services. J Emot Behav Disord. (2011) 19:83–97. 10.1177/1063426609344604

[52] Williams CD Lindsey M Joe S. Parent–adolescent concordance on perceived need for mental health services and its impact on service use. Child Youth Serv Rev. (2011) 33:2253–60. 10.1016/j.childyouth.2011.07.011 22628903 PMC3357129

[53] Yasui M Hipwell AE Stepp SD Keenan K. Psychocultural correlates of mental health service utilization among African American and European American girls. Adm Policy Ment Health Ment Health Serv Res. (2015) 42:756–66. 10.1007/s10488-014-0610-0 25380787 PMC4517982

[54] Yeh M McCabe K Hough RL Lau A Fakhry F Garland A. Why bother with beliefs? Examining relationships between race/ethnicity, parental beliefs about causes of child problems, and mental health service use. J Consult Clin Psychol. (2005) 73:800. 10.1037/0022-006X.73.5.800 16287380

[55] Yeh M Lambros K Tsai K Zerr A Trang D McCabe K. Multistakeholder etiological explanation agreement and adolescent/parent treatment engagement. J Clin Child Adolesc Psychol. (2019) 48:42–53. 10.1080/15374416.2018.1520120 30652924 PMC12413901

[56] Srebnik D Cauce AM Baydar N. Help-seeking pathways for children and adolescents. J Emot Behav Disord. (1996) 4:210–20. 10.1177/106342669600400402

[57] Chang DF Sue S. The effects of race and problem type on teachers' assessments of student behavior. J Consult Clin Psychol. (2003) 71:235. 10.1037/0022-006X.71.2.235 12699018

[58] Leong FT Lau AS. Barriers to providing effective mental health services to Asian Americans. Ment Health Serv Res. (2001) 3:201–14. 10.1023/A:1013177014788 11859966

[59] Froeschle J Moyer M. Just cut it out: legal and ethical challenges in counseling students who self-mutilate. Professional School Counseling. (2004) 7:231–5.

[60] Snowden LR Masland MC Libby AM Wallace N Fawley K. Racial/ethnic minority children's use of psychiatric emergency care in California's public mental health system. Am J Public Health. (2008) 98:118–24. 10.2105/AJPH.2006.105361 18048783 PMC2156049

[61] Sue S Fujino DC Hu LT Takeuchi DT Zane NW. Community mental health services for ethnic minority groups: a test of the cultural responsiveness hypothesis. J Consult Clin Psychol. (1991) 59:533. 10.1037/0022-006X.59.4.533 1918557

[62] American Academy of Child and Adolescent Psychiatry Committee on Health Care Access and Economics Task Force on Mental Health. Improving mental health services in primary care: reducing administrative and financial barriers to access and collaboration. Pediatrics. (2009) 123:1248–51. 10.1542/peds.2009-0048 19336386

[63] Lives H. Brighter Futures: The Strategy for Children and Young People's Health. London: Department for Children, Schools and Families/Department of Health. (2009)

[64] Burns ME Leininger LJ. Understanding the gap in primary care access and use between teens and younger children. Med Care Res Rev. (2012) 69:581–601. 10.1177/1077558712453335 22842583

[65] Knapp PK Foy JM. Integrating mental health care into pediatric primary care settings. J Am Acad Child Adolesc Psychiatry. (2012) 10:982–4. 10.1016/j.jaac.2012.07.009 23021473

[66] Richardson LP McCarty CA Radovic A Suleiman AB. Research in the integration of behavioral health for adolescents and young adults in primary care settings: a systematic review. J Adolesc Health. (2017) 60:261–9. 10.1016/j.jadohealth.2016.11.013 28087267 PMC5973784

